# Hemorrhagic complications associated with suprachoroidal buckling

**DOI:** 10.1186/s40942-020-00211-6

**Published:** 2020-04-16

**Authors:** Fares Antaki, Ali Dirani, Marina Ravagnani Ciongoli, David H. W. Steel, Flavio Rezende

**Affiliations:** 1 Department of Ophthalmology, Hôpital Maisonneuve-Rosemont, Université de Montréal, 5415 Assumption Blvd, Montréal, QC H1T 2M4 Canada; 2 Centre Universitaire d’Ophtalmologie, Hôpital du Saint-Sacrement, CHU de Québec-Université Laval, Québec, QC Canada; 3 Department of Ophthalmology, University of Sao Paulo, Sao Paulo, Brazil; 4 Institute of Genetic Medicine, Newcastle University, Newcastle upon Tyne, UK; 5 Sunderland Eye Infirmary, Sunderland, UK

**Keywords:** Retinal detachment, Suprachoroidal buckling, Viscoelastic buckling, Hemorrhagic complications

## Abstract

**Background:**

Multiple surgical techniques exist for the repair of rhegmatogenous retinal detachments (RRD). Suprachoroidal buckling (SCB), consisting of injecting viscoelastic material in the suprachoroidal space to allow chorioretinal apposition, has been recently described in the repair of RRD. The aim of this study is to report the complications of SCB and to propose measures to decrease their incidence during the learning curve.

**Methods:**

A total of 26 eyes of 26 patients who underwent a SCB procedure for the management of RRD secondary to a single or multiple retinal breaks were enrolled. Patients were operated between January 2014 and March 2017 at two academic institutions. Patient and retinal detachment characteristics were obtained from the charts. Surgical videos were reviewed for every case and intraoperative complications were recorded. Complications observed postoperatively were obtained from the charts.

**Results:**

Sixteen eyes (62%) underwent SCB alone, 5 eyes (19%) underwent additional gas tamponade and 5 eyes (19%) had combined pars plana vitrectomy. The most common complication was hemorrhage (6 cases, 23%). There were no cases of ischemic choroidal changes or hyperpigmentation at the edge of the dome. All six complications occurred in phakic patients who had inferior RRD with retinal breaks in the inferior quadrants. Isolated subretinal hemorrhage occurred in 4 patients and isolated suprachoroidal hemorrhage in 1 patient, and those did not affect final visual outcome. Extensive combined subretinal and suprachoroidal hemorrhage occurred in one case, and was complicated by phthisis bulbi. Re-detachment occurred in 4/6 (67%) of patients, and 5/6 (83%) of patients required a secondary procedure. Three out of 6 patients (50%) had at least 2 lines of visual acuity improvement.

**Conclusion:**

SCB performed for RRD can be associated with hemorrhagic complications. The hemorrhages are usually self-limited but may occasionally result in severe visual compromise when involving the suprachoroidal space. Specific surgical measures need to be undertaken in order to decrease the likelihood of complications and further studies are needed to assess the safety and efficacy of this technique.

## Introduction

Rhegmatogenous retinal detachment (RRD) is the most common form of retinal detachment (RD) and is associated with a high risk of visual impairment and complications if left untreated [1]. Different surgical techniques exist for the repair of RRD and they include: scleral buckling (SB), pars plana vitrectomy (PPV), a combined PPV/SB, and pneumatic retinopexy [2]. Pars plana vitrectomy remains the most commonly used technique and provides very good anatomic success, especially in pseudophakic patients [3]. However, in young phakic patients with RRD associated with inferior retinal breaks/holes, SB is still a highly effective alternative compared to other treatment methods [4]. Yet, SB surgery is associated with risks of induced refractive changes, strabismus, diplopia, chronic ocular pain and other explant-related complications [5, 6].

In 1983, Poole et al. described the use of the space between the choroid and the sclera, the suprachoroidal space rather than the episcleral space for buckling in the treatment of retinal detachment. This approach could mitigate the risks described above [7]. Recently, the opportunity of using this space has been revisited with a novel technique described for suprachoroidal buckling (SCB), consisting of injecting viscoelastic material (e.g. sodium hyaluronate) in the suprachoroidal space, thereby temporarily indenting the choroid at the desired location where peripheral breaks are present to allow chorioretinal apposition and retinal reattachment [8, 9]. Several studies have demonstrated the safety and effectiveness of this technique with single surgery reattachment rates around 90% [10, 11].

Presently, few surgeons have adopted this technique mainly due to concerns regarding hemorrhagic complications. In the literature, data is lacking on the safety of SCB and on methods to decrease the risk these intraoperative events. In this paper, we report complications of SCB and propose adjustments in the surgical technique to improve the safety of this procedure during the surgical learning curve.

## Methods

We reviewed the charts of twenty-six consecutive patients with RRD who underwent a SCB procedure by two surgeons (FR and DS) at two institutions (Hôpital Maisonneuve-Rosemont, CIUSSS de l’Est-de-l’Île-de-Montréal, Université de Montréal, Montréal, Canada; and Sunderland Eye Infirmary, Sunderland, United Kingdom) between January 2014 and March 2017. The decision to perform SCB as an isolated or combined procedure was at the discretion of the operating surgeon. Based on the reported safety and efficacy of SCB in the literature [10], SCB was proposed to patients with non-complicated retinal detachments that were, most of the time, associated to inferior retinal breaks.

Demographic data including sex, gender, age and operated eye were retrieved from the charts. Selected pre-operative examination information were recorded. They included past medical and surgical history, medications, patient’s ophthalmic history, pertinent findings on slit-lamp microscopy (e.g. lens status) and pre-operative best corrected visual acuity (BCVA). Retinal detachment characteristics were noted: involvement of the macula, extent of the detachment, and, type, number and location of retinal breaks. Operative reports and video recordings were reviewed and the steps of the surgical technique were noted for every case. Relevant intraoperative findings were noted including the type of surgery, the type of tamponade (when used) and the type of viscoelastic material used. All intraoperative complications were noted after identification of the surgical steps that preceded the event. Postoperatively, patients were followed for a minimum of 6 months and care was taken to document any postoperative complication such as choroidal ischemic or retinal pigment epithelium (RPE) changes. In the cases of anatomic failure, secondary procedures performed were recorded and final outcomes were retrieved from the chart using the most recent ophthalmologic examination.

### Surgical technique

**Fig. 1:**
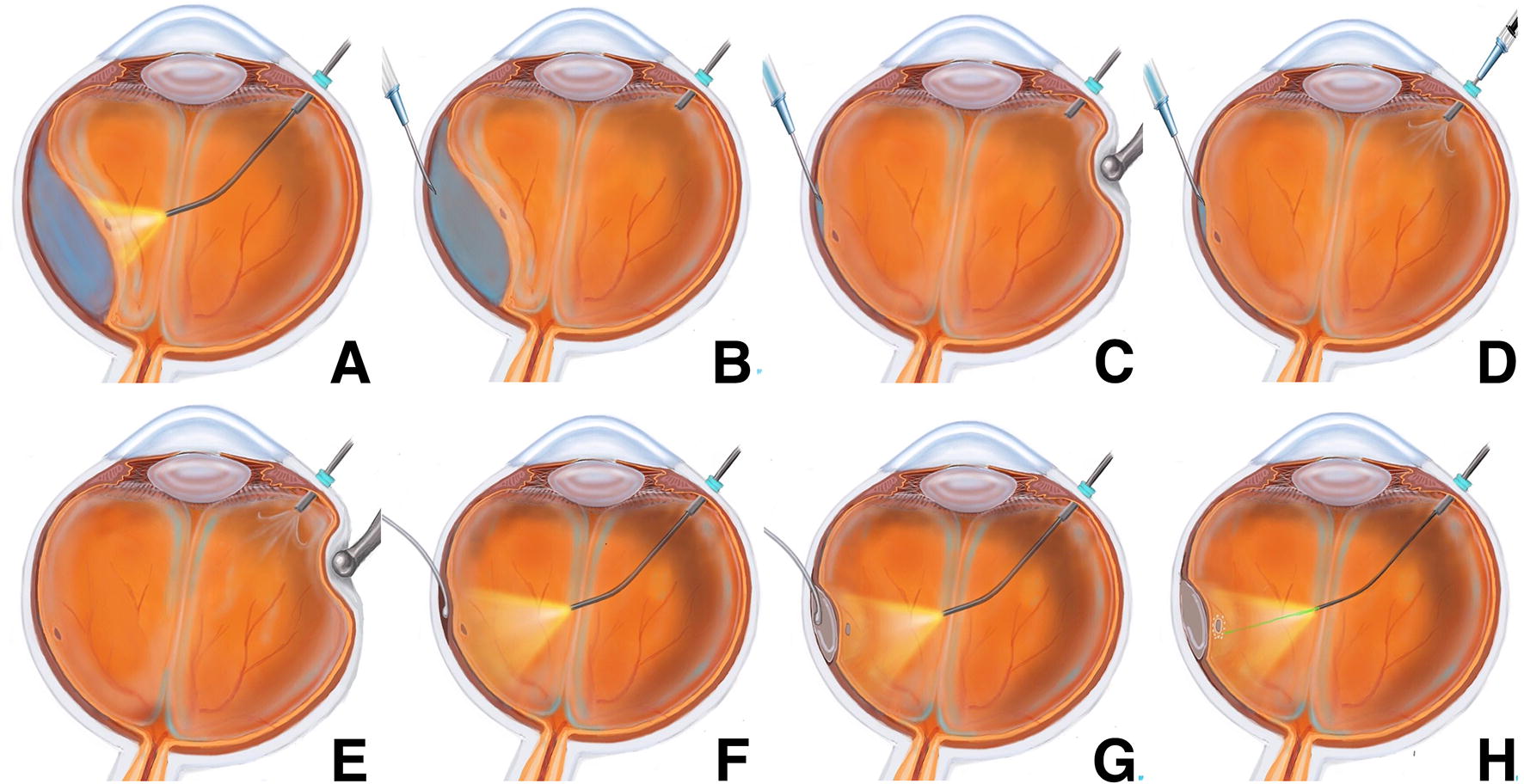
Step-by-step illustration of the suprachoroidal buckling procedure. **a** Under direct visualization using a wide-angle viewing system, the extent of the detachment and the localization of the breaks is assessed. **b** Subretinal fluid drainage is done under direct visualization using a 26-gauge needle mounted on a 3-ml syringe (without plunger). **c**–**e** A pressure on the sclera is carried using a scleral depressor at the same time of drainage to avoid ocular hypotony. **f**, **g** An olive-tipped 20-gauge cannula is used to inject the viscoelastic material. The cannula is guided through the suprachoroidal space and directed posteriorly towards the site of the break under visualization through the wide-field viewing system. The viscoelastic is injected to create the desired dome effect. **h** Under direct visualization, endolaser retinopexy is performed around the identified breaks

A meticulous preoperative examination is performed to determine the extent of detachment, the number and location of all retinal breaks. A 25-gauge valved trocar is first inserted superotemporally or superonasally 4 mm from limbus, ideally 180° from the original retinal breaks for better visualization of the area to be treated. Fundus examination is performed using a wide-angle viewing system (BIOM noncontact panoramic viewing system; Oculus Surgical, Wetzlar, Germany). The extent of the detachment and the localization of the breaks are assessed using scleral depression and a curved illuminated laser probe transvitreally (without performing vitrectomy) (Fig. 1a).

A conjunctival peritomy is then performed in the quadrant of the located break(s). If the detached area is greater than 2 clock hours or if the detachment is chronic, drainage of subretinal fluid (SRF) is carried out with a 26-gauge needle attached to a 3 cc syringe containing little balanced salt solution (BSS), without the plunger. The needle is introduced bevel down, facing the RPE, to avoid retinal incarceration. It is done as posterior as possible to avoid the vortex veins ampulla. During the resultant passive drainage, scleral depression is performed to increase intraocular pressure (IOP), encouraging egress of SRF and reducing the chance of hemorrhage. After drainage, the needle is removed but the scleral depression is maintained to avoid hypotony. BSS can be injected through the valved trocar at the pars plana or in the anterior chamber to reestablish the IOP (Fig. 1b–e).

Subsequently, a 7.0 Vicryl (Ethicon, LLC, Somerville, NJ) mattress suture is preplaced: parallel to the limbus at the site of the planned sclerotomy (4 mm), with one pass just posterior (5 mm) and another just anterior (3 mm) to the sclerotomy site. A full thickness sclerotomy is then carried out using a crescent knife until complete exposure of the uveal tissue at the pars plana. Careful diathermy during the dissection can help open the slit and improve visualization of the choroid. Viscoelastic material (Healon 5, Abbott Medical Optics) is initially injected only at the posterior lip of the sclerotomy. This creates a small pocket in the suprachoroidal space that allows us to then separate the uvea from the sclera using the cannula that comes in the Healon 5 package (27-gauge Rycroft cannula). Next, an olive-tipped 23-gauge curved cannula is used to inject the viscoelastic, mounted with a silicone tube on the Healon 5 syringe (El-Rayes curved suprachoroidal cannula; MedOne Surgical, Sarasota, FL). The cannula is advanced into the suprachoroidal space and directed posteriorly towards the site of the retinal break under microscope visualization, using the light of the curved illuminated laser probe. The cannula is introduced in a way by which the olive tip faces the sclera while the curved cannula shaft indents the choroid (i.e. the opposite direction to that the surgeon may intuitively choose). When progressing into the suprachoroidal space, the olive tip and the cannula shaft are identified as a choroidal indentation as they are moved nasally/temporally or posteriorly to reach the desired location (Fig. 1f). The viscoelastic is then injected: first, posterior to the most posterior retinal break, displacing the choroid and creating a dome that produces the desired buckling effect. As Healon 5 is injected, the cannula is moved more anteriorly or laterally. At this time, more viscoelastic injected until the retinal break(s) is (are) entirely covered by the buckling effect (Fig. 1g). The cannula is then withdrawn and sclerotomy closed by tightening the preplaced suture. Transvitreal endolaser retinopexy is performed around the identified retinal breaks over the indented flat retina (Fig. 1h). At the end of the procedure, the incised conjunctiva is closed with 7.0 Vicryl and the trocar is removed.

For cases combined with PPV, a 25-gauge vitrectomy system is used (Constellation, Alcon, ForthWorth, TX, USA)). Core vitrectomy is performed followed by the induction of posterior vitreous detachment if needed. Vitreous base shaving is performed with scleral indentation. SRF is drained first, either using perfluorocarbon liquid or direct fluid-air exchange (FAX) draining through the primary retinal break. Suprachoroidal buckling would then be carried out. Further drainage through the indented breaks was carried out only in the early cases. After encountering complications during this step of the procedure, we decided to never drain breaks following SCB. Gas exchange would then be performed according to the surgeon’s preference (air, sulfur hexafluoride SF_6_, octafluoropropane C_3_F_8_ or silicone oil).

## Results

Twenty-six patients underwent a SCB procedure between January 2014 and March 2017. Baseline patient and study eye characteristics are summarized in Table 1. The median age was 54 (IQR = 14) and most patients had no prior ocular history, 21/26 (81%). Almost all patients were phakic, 25/26 (96%), and most had retinal detachments associated to inferior retinal breaks, 24/32 breaks (75%).

**Table 1:** Baseline patient and study eye characteristics from consecutive patients who underwent suprachoroidal buckling between 2014 and 2017

Variable	Total cohort (n = 26)
Age, median (IQR)	54 (14)
Male gender, n (%)	15 (58)
Past ocular history excluding cataract surgery, n (%)	
None	21 (81)
Previous pars plana vitrectomy	0 (0)
Failed pneumatic retinopexy for current retinal detachment	5 (19)
Preoperative lens status, n (%)	
Phakic	25 (96)
Pseudophakic	1 (4)
Retinal detachment characteristics, n (%)	
Retinal detachment macular status	
Macula-on	11 (42)
Macula-off	15 (58)
Number of clock hours of retinal detachment	
Median (IQR)	4 (3)
Mean (SD)	4.4 (1.5)
Range	1–7
Retinal breaks characteristics, n (%)	
Number of breaks in the detached retina	
None visible	2 (8)
1	17 (65)
2	4 (15)
3	3 (12)
Median (IQR)	1 (1)
Type of breaks in the detached retina (n = 34, total retinal breaks)	
None visible	2 (6)
Horseshoe tear	23 (68)
Dialysis	3 (8)
Hole	6 (18)
Location of the retinal break (n = 32, visible breaks)	
12-o’clock meridian	0 (0)
11- or 1- o’clock meridians	0 (0)
10- or 2- o’clock meridians	6 (19)
9- or 3- o’clock meridians	2 (6)
8- or 4- o’clock meridians	13 (41)
7- or 5- o’clock meridians	10 (31)
6- o’clock meridian	1 (3)

Table 2. summarizes the operative parameters of the SCB procedure. SCB alone was performed in 16/26 cases (62%), SCB with gas tamponade in 5/26 cases (19%) and SCB combined to pars plana vitrectomy and tamponade in 5/26 cases (19%). Perfluoropropane (C_3_F_8_) was the most commonly used tamponade agent, 6/10 (60%).

**Table 2:** Surgical parameters in the suprachoroidal buckling procedures

Procedure	Total cohort (n = 26)
Suprachoroidal buckling alone, n (%)	16 (62)
Suprachoroidal buckling with gas tamponade, n (%)	5 (19)
Tamponade	
Perfluoropropane (C_3_F_8_)	4 (80)
Sulfur hexafluoride (SF_6_)	1 (20)
Suprachoroidal buckling with pars plana vitrectomy, n (%)	5 (19)
Tamponade	
Air	1 (20)
Perfluoropropane (C_3_F_8_)	2 (40)
Sulfur hexafluoride (SF_6_)	0 (0)
Silicone oil	2 (40)

Six complications were encountered in the cohort (23%), all of which occurred between March 2014 and June 2015, during the first year of the implementation of this technique. The complicated cases were part of the first ten consecutive eyes that underwent SCB. In the subsequent 13 cases, there were no complications. The six complications were hemorrhagic in nature. In parallel, we looked for the presence of ischemic choroidal changes and hyperpigmentation at the edge of the created choroidal indentation as potential complications of SCB. These complications were not observed in our cohort (Table 3).

**Table 3:** Rate of complication per suprachoroidal buckling procedure

Variable	SCB alone (n = 16)	SCB + gas (n = 5)	SCB + PPV (n = 5)	Overall (n = 26)
Type of complications, n (%)				
Intraoperative or postoperative hemorrhage	3 (19)	2 (40)	1 (20)	6 (23)
Hyperpigmentation at the edge of the indentation	0 (0)	0 (0)	0 (0)	0 (0)
Ischemic choroidal changes	0 (0)	0 (0)	0 (0)	0 (0)

*SCB* suprachoroidal buckling, *PPV* pars plana vitrectomy

All six patients who developed hemorrhagic complications following SCB were phakic with inferior RRD and identified retinal breaks (tear, hole or dialysis) in the inferior quadrants (Table 4). Among those patients, three were undergoing SCB alone, two others were planned for SCB with tamponade (SF_6_ and C_3_F_8_) and the last patient was scheduled to receive a combined procedure of SCB, PPV and cataract extraction.

**Table 4:** Demographic and retinal detachment characteristics for patients with hemorrhagic complications

Case, eye	Demographics		PM/Ochx	Retinal detachment (RD)				BCVA	Other
	Age, sex	Lens status		Macular involvement	RD extent (clockwise clock hours)	Number of breaks	Break location (clock hour)		
1, OD	29, F	Phakic	High myopia (− 18.00D)	OFF	3–9	1	Hole at 4	20/60	
2, OD	62, M	Phakic	Depression on trazodone; osteoarthritis on celecoxib; Macula OFF RD, s/p pneumatic retinopexy failure	ON	5–8	1	Tear at 8	20/50	
3, OD	28, F	Phakic	–	OFF	3–9	1	Dialysis 5–7	20/70	
4, OS	52, F	Phakic	Hypertension	ON	4–7	1	Tear at 4	20/20	
5, OS	60, F	Phakic	Cardiovascular disease on aspirin	OFF	3–10	1	Tear at 7	CF	VH
6, OD	65, M	Phakic	–	ON	6–8	1	Tear at 7	20/20	VH

*PM/Ochx* past medical and ocular history, *VH* vitreous hemorrhage, *BCVA* best-corrected visual acuity (pre-operative in this case), *RD* retinal detachment, *CF* counting fingers

Table 5 highlights the details of the hemorrhagic complications. Four out of 6 (67%) patients had isolated subretinal hemorrhage, one patient had an isolated suprachoroidal hemorrhage and one had a combined subretinal and suprachoroidal hemorrhage. All areas of hemorrhage were inferior, adjacent to the site of the tears. Four out of 6 (67%) hemorrhages were identified intraoperatively. Two occurred during external fluid drainage, one during the injection of the viscoelastic material and another one occurred during fluid- air exchange (FAX), during PPV. One of the hemorrhages identified postoperatively was localized to the site of external fluid drainage. The final one was localized to the area of choroidal indentation and was near a vortex ampulla (Table 5).

**Table 5:** Description of the hemorrhagic complications and final outcomes

Case/Eye	Procedure	Hemorrhagic complication					Post-operative course			Final outcomes	
		Level	Timing	Step	Location	Site	Post-op outcomes	Associated post-op finding	Secondary procedure	Final anatomic status	Final BCVA
1 OD	SCB	Subretinal	Intra-operative	External fluid drainage	Inferonasal	Adjacent to the tear	Failure at day 1		PPV + C_3_F_8_	Success	20/40 (PSCC)
2 OD	SCB	Subretinal	Intra-operative	Injection of Healon 5 in the suprachoroidal space	Inferotemporal	Adjacent to the choroidal indentation (dome)	Success	BCVA 20/40 with 2 + NS and mild VH	Phaco + IOL + PPV and peeling for ERM at 1 year	Success	20/25
3 OD	SCB + C_3_F_8_	Subretinal	Intra-operative	External fluid drainage	Inferonasal with posterior extension through a gutter to the macula	At the site of the external fluid drainage	Failure at week 1		PPV + + Phaco + IOL + C_3_F_8_	Success	20/50
4 OS	SCB + SF_6_	Subretinal	Post-operative	–	Inferotemporal	At the site of the subretinal fluid drainage	Failure at week 1	Small and localized subretinal hemorrhage	PPV + Phaco + IOL + C_3_F_8_	Success	20/25
5 OS	SCB + PPV + Phaco + IOL + silicon oil	Subretinal + suprachoroidal	Intra-operative	FAX	Inferior (180 degrees)		Failure at month 2	Post-op hyphema	PPV + FAX + removal of IOL and bag + silicon oil	Hypotony, advanced PVR and phthisis bulbi	NLP
6 OD	SCB	Suprachoroidal	Post-operative	–	Inferotemporal	Adjacent to the choroidal indentation (dome)	Success	Mild VH (seen pre-op)	None	Success	20/30

*AC* anterior chamber, *C*_*3*_*F*_*8*_ isolated octafluoropropane (C_3_F_8_) intravitreal gas injection, *ERM* epiretinal membrane, *Failure* anatomic failure defined as post-operative re-detachment of the retina, *FAX* fluid-air exchange, *IOL* intraocular lens, *NS* nuclear sclerosis, *PPV* par plana vitrectomy, *Phaco* phacoemulsification, *PSCC* posterior subcapsular cataract, *Success* anatomic success defined as post-operative attached retina, *SF*_*6*_ isolated sulfur hexafluoride (SF_6_) intravitreal gas injection, *SCB* suprachoroidal buckling, *VH* vitreous hemorrhage

Anatomic failure, defined as the recurrent detachment of the retina, was seen in 4 out of 6 patients (67%) after the primary planned procedure. Five out of 6 (83%) patients required a secondary surgery that included a PPV, with or without tamponading agent and sometimes combined with a phacoemulsification procedure. One patient underwent PPV due to a breakthrough persistent vitreous hemorrhage. After secondary surgery, anatomic success was seen in 4 out of 5 patients (80%). The remaining patient had a recurrent retinal detachment and advanced proliferative vitreoretinopathy (PVR), eventually leading to phthisis bulbi.

Visual outcomes were as follows: in patients with macula-off retinal detachments, a BCVA improvement of two lines was seen in patients 1 and 3. However, in case 5, severe vision loss was observed (no light perception, NLP).

In patients with macula-on retinal detachments, a BCVA improvement of two lines was noted in case 2. However, patients 4 and 5 lost one line of BCVA. Herewith, we present a detailed description of the cases with hemorrhagic complications (Additional file 1):

### Case 1

**Fig. 2:**
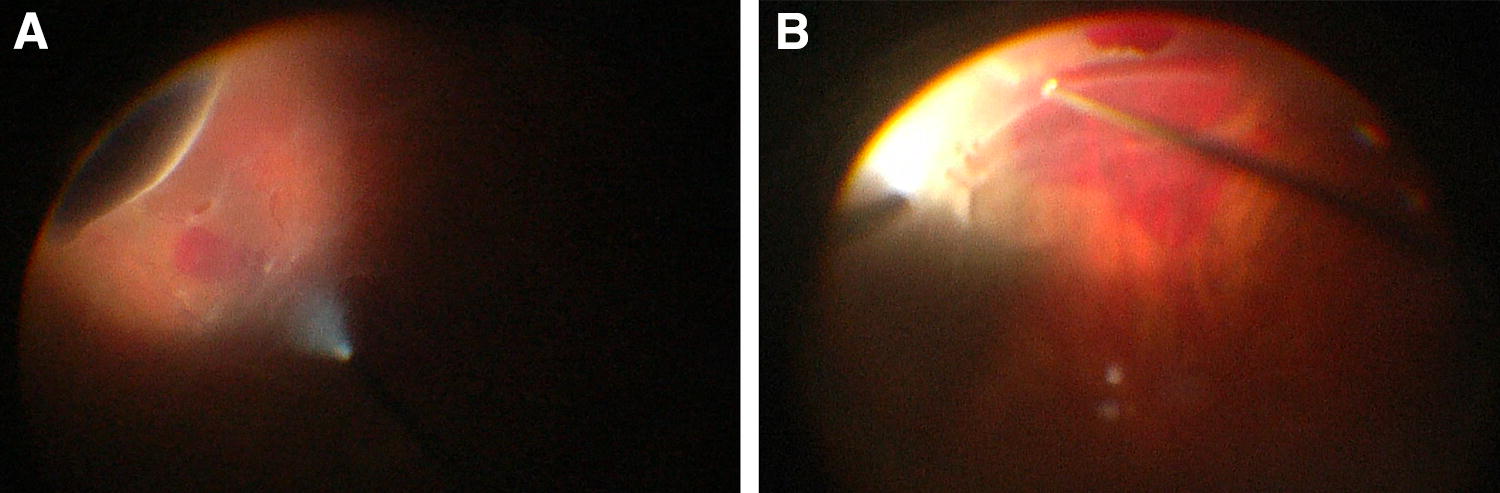
Case 1. **a** Operative findings. Photo taken during the primary surgery at the time of external subretinal fluid drainage using a 26-gauge needle. It shows an inferonasal subretinal hemorrhage adjacent to the causative tear. **b** Operative findings. Photo taken during the secondary surgery. It shows re-bleeding from the tear leading to a localized subretinal hemorrhage

A 29-year-old phakic woman with high myopia (− 18.00D OD) presented with a macula-off retinal detachment extending from 3-o’clock to 9-o’clock. A hole was identified at 4-o’clock. The patient’s BCVA was 20/60. Isolated SCB was performed. Inferonasal subretinal hemorrhage occurred adjacent to the causative tear. This happened during the external subretinal fluid drainage using a 26-gauge needle. Postoperative day 1 examination revealed anatomic failure with an inferior retinal detachment. A secondary PPV with injection of C_3_F_8_ was performed. During this second procedure, rebleeding from the tear occurred leading to a localized subretinal hemorrhage (Fig. 2). Postoperatively, the retina was attached. At 18 months, the patient’s BCVA was 20/40 with a posterior subcapsular cataract (PSCC).

### Case 2

**Fig. 3:**
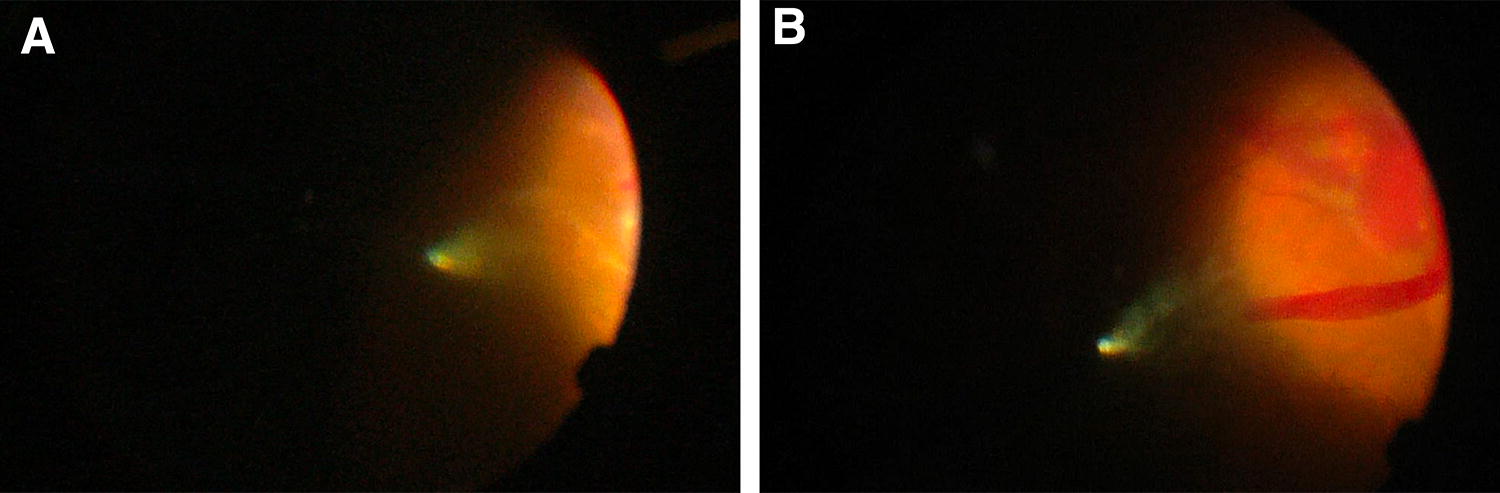
Case 2. **a** Operative findings. Photo taken under direct visualization of the injection of viscoelastic material (Healon 5) in the suprachoroidal space using the cannula. It shows the faint beginning of a subretinal hemorrhage. **b** Operative findings. Photo taken after the beginning of the subretinal hemorrhage. It shows expansion of the hemorrhage inferotemporally, adjacent to the dome of the indentation created by the injection

A 62-year-old phakic man with a past medical history of depression treated with trazodone and osteoarthritis treated with regular celecoxib underwent a SCB procedure. He has a past ocular history of failed pneumatic retinopexy for a macula OFF retinal detachment. In this case, the macula was attached and the detachment spanned three clock hours from 5-o’clock to 8-o’clock. One visible tear was seen at 8-o’clock. The BCVA was 20/50. The surgery was complicated by inferotemporal subretinal hemorrhage, adjacent to the location of the choroidal indentation created by the SCB (Fig. 3). This occurred during injection of the viscoelastic material. Postoperatively, day 1 examination revealed mild vitreous hemorrhage. Anatomic success was achieved and the BCVA was 20/40 (with 2 + NS). After 1 year, cataract extraction and epiretinal membrane (ERM) peeling was performed. At the last follow-up visit, 6 months postoperatively, the retina was attached and the visual acuity was 20/25.

### Case 3

**Fig. 4:**
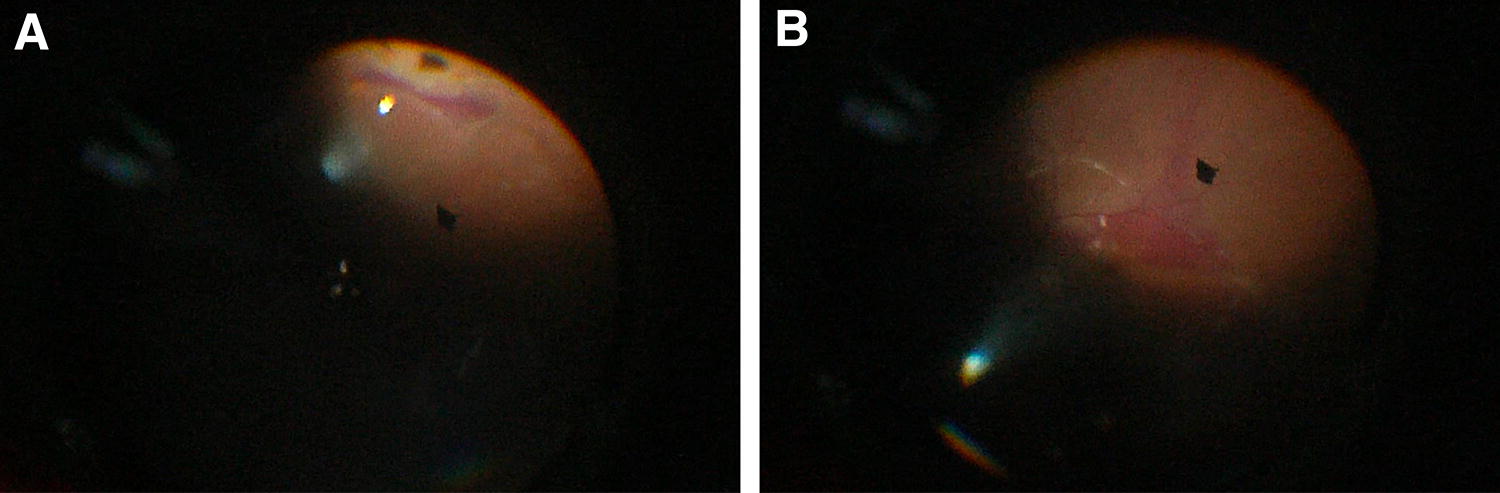
Case 3. **a** Operative findings. Photo taken during the external subretinal fluid drainage using a 26-gauge needle. It shows the beginning of a subretinal hemorrhage. **b** Operative findings. Photo taken after the beginning of the subretinal hemorrhage. It shows expansion of the hemorrhage posteriorly, through a subretinal gutter and reaching the macula

A 28-year-old phakic woman with no past ocular history presented with a macula-off retinal detachment from 3-o’clock to 9-o’clock with retinal dialysis from 5-o’clock to 7-o’clock. The BCVA was 20/70. The patient underwent SCB with intravitreal gas injection of C_3_F_8_. The hemorrhage occurred during the external subretinal fluid drainage using a 26-gauge needle. The hemorrhage extended posteriorly towards the fovea (Fig. 4). The 1-week examination, re-detachment was noted. The patient underwent a secondary procedure consisting of PPV, phacoemulsification with IOL implantation and C_3_F_8_ injection. At the last follow-up visit, the retina remained attached and BCVA was 20/50.

### Case 4

A 52-year-old phakic woman with known treated hypertension and no past ocular history presented with a macula ON retinal detachment from 4-o’clock to 7-o’clock. A tear was identified at 4-o’clock. The BCVA was 20/20. SCB with SF_6_ intravitreal gas injection was performed. The surgery was uneventful. During postoperative visit at day 1, a small localized hemorrhage was noted inferotemporally at the site of the subretinal fluid drainage. At 1 week postoperatively, re-detachment was noted. A secondary PPV with C_3_F_8_ and phacoemulsification and IOL was performed. At the last follow-up visit, the retina remained attached and BCVA was 20/25.

### Case 5

**Fig. 5:**
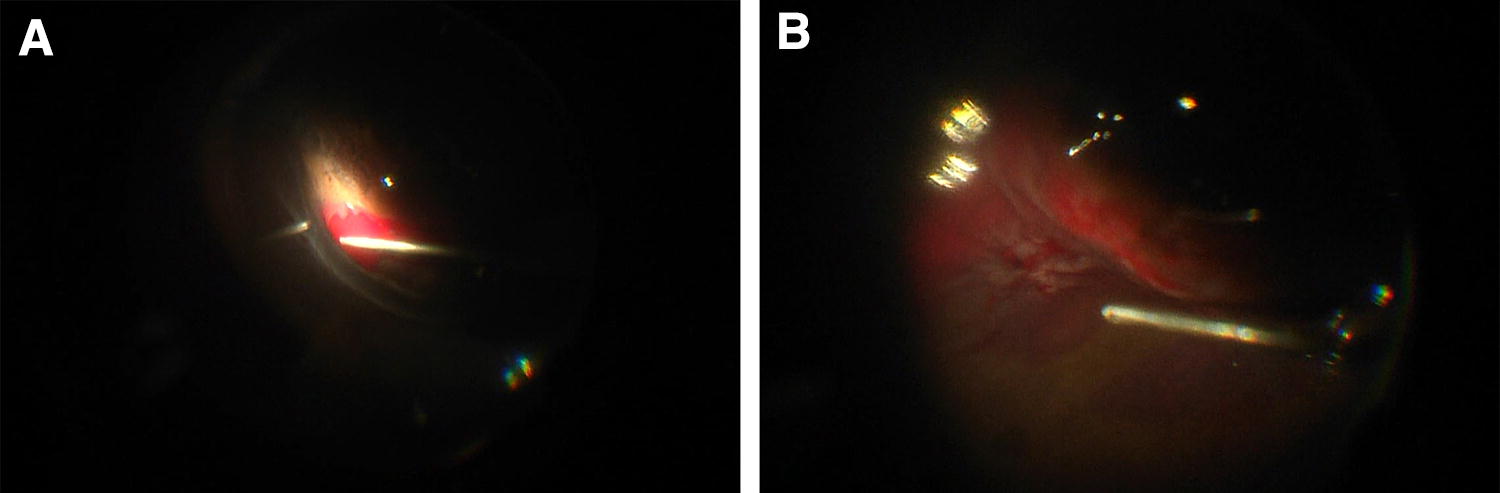
Case 5. **a** Operative findings. Photo taken during fluid-air exchange (FAX) while aspirating subretinal fluid. It shows the beginning of a hemorrhage occurring at the dome created by the suprachoroidal buckling (SCB). **b** Operative findings. Photo taken during an attempt to drain subretinal hemorrhage through an inferonasal retinotomy. It shows diffuse inferior subretinal hemorrhage with a possible suprachoroidal component, spanning 180 degrees and sparring the macula

A 60-year-old phakic woman with known cardiovascular disease on aspirin and no past ocular history presented to us with a macula-off retinal detachment spanning seven clock hours from 3-o’clock to 10-o’clock. A tear was identified at 7-o’clock. 2 + vitreous hemorrhage was noted on examination and BCVA was counting fingers (CF). SCB, PPV and phacoemulsification with intraocular lens implantation was planned. The patient was asked to discontinue the use of aspirin 2 days prior to surgery. Intraoperatively, during FAX, while aspirating subretinal fluid, hemorrhage occurred from the border of the tear at the dome created by the SCB and extended subretinally and suprachoroidally spanning 180 degrees but sparing the macula (Fig. 5). A posterior retinotomy was performed inferonasally and an attempt was made to drain the subretinal hemorrhage. At the end of the procedure, silicone oil was used as tamponade. Postoperatively, a hyphema was noted. The patient was followed, and partial resolution of the subretinal hemorrhage was noted. At 2-months postoperatively, the patient presented with recurrent detachment and advanced proliferative vitreoretinopathy (PVR). A secondary procedure was performed consisting of PPV, FAX, removal of the intraocular lens and bag and silicone oil tamponade. During the surgery, subretinal fluid and hemorrhage could not be completely drained. On follow-up, hypotony developed progressively eventually leading to phthisis bulbi at 1 year.

### Case 6

**Fig. 6:**
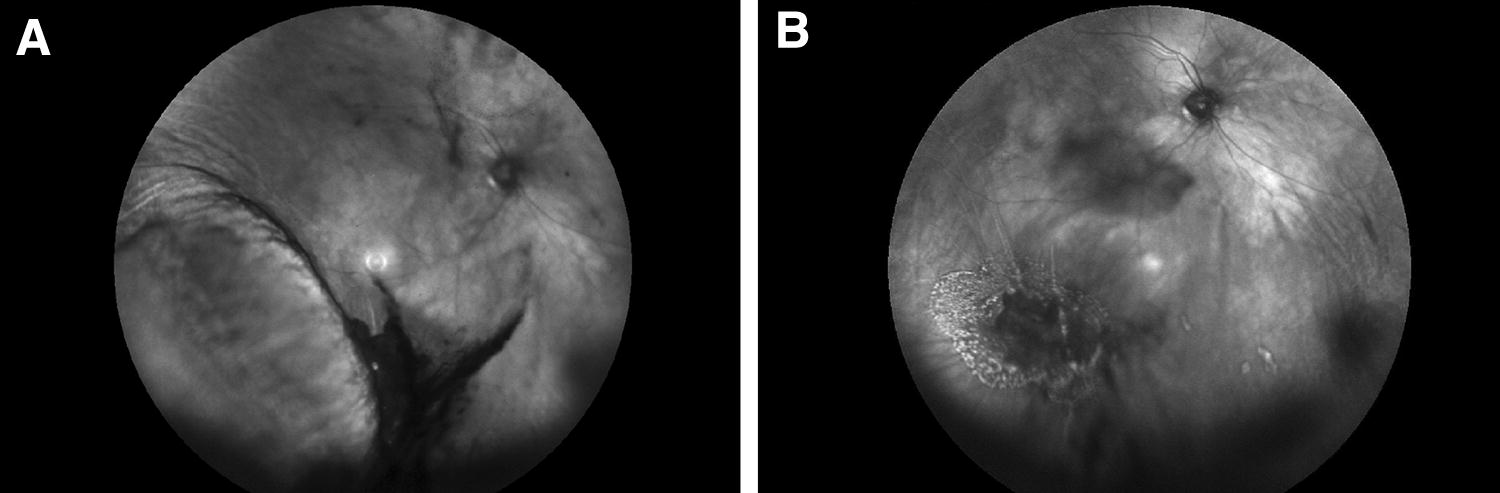
Case 6. **a** Red-free fundus photograph taken on postoperative day 2. It shows inferotemporal suprachoroidal hemorrhage, and vitreous hemorrhage that was noted preoperatively. **b** Red-free fundus photograph taken on postoperative week 6. It shows an attached retina and old vitreous hemorrhage gradually resolving

A 65-year old phakic man with no past ocular history presented with a macula-on retinal detachment spanning two clock hours from 6-o’clock to 8-o’clock. A large posterior and inferior tear was identified at 7 o’clock with shallow subretinal fluid. Mild (1+) vitreous hemorrhage was noted on examination and BCVA was 20/20. The patient was planned for isolated SCB. Intraoperatively, cryotherapy was performed to the tear and no subretinal fluid drainage was done. There were no intraoperative complications. On day 1 follow-up, inferotemporal suprachoroidal haemorrhage was seen (Fig. 6). The intraocular pressure (IOP) was 55 mmHg and corneal oedema was present. The patient was treated with intravenous acetazolamide. The patient was followed and the vision gradually improved: at 3 months, the old vitreous and suprachoroidal haemorrhages slowly resolved and the retina remained attached. No secondary procedure was required and BCVA was 20/30.

## Discussion

With the rise of vitrectomy in the treatment of RRD, trainee surgeons are getting less exposure to scleral buckling surgery. Nonetheless, in phakic eyes with atrophic hole-related or dialysis-related RRD without posterior vitreous detachment, SB is still the preferred option by most surgeons. This is especially true if the breaks are located inferiorly. In the past, many surgeons have described the use of vitrectomy instruments for SB, including chandelier lighting and wide-angle viewing systems on surgical microscopes [12, 13]. Similarly, the development of new techniques to drain choroidal detachments, using trocars placed in the suprachoroidal space, have allowed surgeons to be more familiarized with this region of the posterior segment [14, 15]. SCB uses techniques that are more familiar to vitrectomy-trained surgeons, but many are still reticent to try it. This is probably due to the fear of entering the suprachoroidal space and the potential complications associated to that.

In this study, we report complications of SCB and describe ways to mitigate the intraoperative risks associated with this procedure. The complications were all hemorrhagic in nature and occurred in six patients, during the surgical learning curve. They were self-limited with no visual consequences in the majority of cases. The hemorrhage occurred mostly during external fluid drainage (cases 1, 3, and 4) or during injection of viscoelastic material in the suprachoroidal space (case 2). Only one case had a severe hemorrhage that led to severe visual loss. In this specific case that underwent combined SCB and PPV, the subretinal and suprachoroidal hemorrhage occurred during FAX and the attempt to drain subretinal fluid through the indented break. The visual and anatomic outcomes were poor, and the patient had hypotony and phthisis bulbi (case 5). None of our cases had massive suprachoroidal hemorrhage (kissing suprachoroidal hemorrhage).

El Rayes et al. first described their surgical technique of SB in 2013 for the treatment of RRD and myopic traction maculopathy. They used a fine bore flexible catheter to inject the hydrogel to create the SCB effect (El-Rayes FlexTip Catheter, MedOne Surgical, Sarasota, FL, USA). No hemorrhagic complications were reported. Two cases of hyperpigmentation at the edge of the indentation were mentioned. They were believed to be due to RPE clumping at the edge of the dome that was created by choroidal indentation. The hyperpigmentation resolved with time. No ischemic choroidal changes at the site of choroidal indentation were noted [9]. In our cohort, no RPE or choroidal changes were observed.

In 2014, El Rayes published 1-year data on the management of 23 patients with myopic vitreoretinal interface disorders again using the SCB technique with the same catheter to reach the posterior pole. There were two cases of choroidal hemorrhage intraoperatively, both in patients with an axial length greater than 33 mm. The first case was in a patient with myopic foveoschisis: the hemorrhage was small, located outside the arcades and was controlled intraoperatively by increasing IOP. The second case occurred in a patient with a myopic macular hole while the catheter was being advanced across the edge of a staphyloma. When the suprachoroidal filler injection was performed, the dome displaced the blood away from the foveal area following tissue plasminogen activator injection [16].

More recently, Mikhail et al. reported one case of limited suprachoroidal hemorrhage in a cohort of 55 eyes treated with the same technique for RRD secondary to peripheral retinal breaks. The complication occurred intraoperatively in a phakic patient with an inferior RRD undergoing SCB alone. The hemorrhage was attributed to the misplacement of the catheter too posteriorly. There was anatomic failure and the patient required a PPV with successful retinal reattachment. The hemorrhage resolved and the final visual acuity was 20/40 [11].

In another retrospective cohort study that included 41 eyes with RRD secondary to peripheral breaks utilizing the same cannula used in the current series, El Rayes reported two patients who had localized suprachoroidal hemorrhages. They occurred intraoperatively at the sclerotomy site and resolved without intervention. There were no visual consequences, and anatomical reattachment was obtained in both cases [10].

In SCB, there is a potential increased risk of suprachoroidal hemorrhage (SCH) due to the suprachoroidal space being contiguous with the highly vascularized choroid. However, it seems that this complication is rather infrequent and rarely affects the surgical outcome. The occurrence of hemorrhagic complications may be related to a variety of different factors. These include ocular risk factors such as high myopia and patient-specific systemic conditions such as hypertension, blood dyscrasia and anticoagulant/antiplatelet use. Finally, there are several intraoperative risk factors for hemorrhage that can be potentially ameliorated by surgical technique including. Those include: ocular hypotony (related to subretinal fluid drainage) and direct injury to the retinal and/or choroidal vascular structures during SCB or drainage.

In case 1, subretinal hemorrhage was self-limited and occurred in a young woman with high myopia and no other risk factors. There was no suprachoroidal component despite her longer axial length, which is a risk factor for SCH during ocular surgery [17]. Similar to cases 3 and 4, the hemorrhage was thought to be associated to the external fluid drainage surgical step. In case 5, the hemorrhage was seen during FAX at the border of the dome created by the SCB, possibly due to instrument touch to the choroid. These techniques are not exclusive to SCB but we believe that the manipulation of the suprachoroidal space during the injection of viscoelastic could increase the risk of bleeding during external drainage and FAX. In case 6, the isolated suprachoroidal hemorrhage was identified postoperatively. The location of the hemorrhage being in proximity with the vortex ampulla suggests a possible direct intraoperative injury to this highly vascularized space. The hemorrhage could also be due to the fact that cryotherapy of the tear was performed prior to viscoelastic injection. Cryotherapy causes choroidal swelling and vascular congestion, and, subsequent indentation and viscoelastic injection could have precipitated the bleed.

In case 2, the complication occurred in a man that was taking trazodone and celecoxib perioperatively for depression and osteoarthritis. He suffered a localized subretinal hemorrhage. Trazodone is an antidepressant that functions as a serotonin antagonist and reuptake inhibitor (SARI) which has been associated with an increased risk of bleeding during surgical procedures [18, 19]. Celecoxib, despite being a non-steroidal anti-inflammatory drug (NSAID), is a highly selective cycloxygenase-2 (COX-2) inhibitor and is not associated with increased risk of intraoperative or postoperative hemorrhage [20]. In case 5, the complication included subretinal and suprachoroidal hemorrhage. The patient was taking aspirin for cardiovascular disease and was asked to discontinue usage 2 days prior to surgery. Recommendations for management of antiplatelet therapy prior to vitreoretinal and retinal detachment surgery show that aspirin is not a major risk factor of hemorrhagic complications intraoperatively [21, 22]. However, risk factors for SCH during PPV include the use of aspirin or warfarin in addition to male sex, advanced age, RRD, the use of a scleral explant and a dropped lens fragment. The incidence of SCH during PPV is reported to be 1% with around 7% of eyes complicated by phthisis bulbi during follow-up [23].

Some of the surgical steps were modified to allow for better visualization and to potentially decrease the risk for hemorrhagic complications. These steps may provide vitreoretinal surgeons with better tools to avoid hemorrhagic complications during the SCB learning curve: (1) using a curved illuminated endolaser probe rather than a chandelier light allows for better control of the eye, and for focal illumination for improved visualization during subretinal fluid needle drainage and suprachoroidal Healon 5 injection. It also allows for laser treatment to be performed around the breaks immediately after subretinal fluid drainage. Many vitreoretinal surgeons are reluctant to use intraocular instruments in the vitreous cavity on a nonvitrectomized eye, but as long as a valved trocar is used for introduction of the endolaser probe, we have noted no complications related to its usage; (2) in chronic or bullous detachments, subretinal fluid drainage is recommended. Otherwise, the viscoelastic may resorb before the subretinal fluid, resulting in primary surgical failure. A 26-gauge needle was used as previously described by Charles [24]. The shaft of the needle is pushed against the sclera first to achieve choroidal indentation and to better expose the choroidal blood vessels through the detached retina. Then, the tip of the needle is inserted into the subretinal space, avoiding larger blood vessels. If at the end of drainage, a small bleed is seen, immediate endolaser can be applied to cauterize it under the flattened retina; (3) the introduction of the olive tip cannula in the direction opposite to its natural curvature is also judged to be safer because the tip is always maintained away from the choroid/retina, avoiding inadvertent trauma to these layers; (4) at the end of the viscoelastic injection, the preplaced suture is important to decrease the outflow of viscoelastic material and to avoid having to pass a needle near a potential uveal prolapse through the sclerotomy.

## Conclusions

In this study, we report the complication rate associated with suprachoroidal buckling technique (23%). We described all six hemorrhagic complications and attempted to identify patient risk factors and intraoperative parameters that might have contributed to those complications. Our study was limited in the aspect that our aim was not to report functional or anatomic outcomes of SCB for RRD. Rather, we sought to describe the complications of SCB and propose an improved surgical technique to increase the safety of SCB. While this procedure has been described in the literature to provide promising outcomes in RRD, in light of the herein reported complications, we believe that this technique should be reserved for retinal detachments related to pathology within the retina rather than the vitreoretinal interface. Possible indications might include: atrophic hole-related RD, RD secondary to retinal dialysis and retinoschisis-related RD. SCB could also be beneficial in post-refractive surgery patients to avoid the hyperopic shift associated with scleral buckling, as well as in phakic patients to prevent post-vitrectomy cataracts. Further studies, that take into account the improved surgical measures highlighted in this paper, are needed to assess the safety and efficacy of this technique.

## Supplementary information

**Additional file 1.** Video montage showing intraoperative footage from cases 1, 2, 3 and 5.

## Data Availability

Not applicable.
